# Musculoskeletal Health Risks Associated With Smartphone Use: A Retrospective Study from Riyadh, Saudi Arabia

**DOI:** 10.7759/cureus.63446

**Published:** 2024-06-29

**Authors:** Jalal Abu Halimah, Mohammed Mojiri, Sultan Hakami, Osama Mobarki, Saleh Alanazi, Ahmad Alharbi, Faisal Khalban, Abdullah Al Sheef, Saleh Alnujaidi, Mohammed Almalawi, Fahad Nasser, Abdulaziz Kreshan, Rawan Almarwani, Salem Ayyashi

**Affiliations:** 1 Department of Surgery, Jazan University, Jazan, SAU; 2 College of Medicine, Jazan University, Jazan, SAU; 3 College of Medicine, Prince Sattam Bin Abdulaziz University, Al-Kharj, SAU; 4 College of Medicine, Ibn Sina National College, Jeddah, SAU; 5 College of Medicine, King Khalid University, Abha, SAU; 6 College of Nursing, University of Hafr Albatin, Hafr Albatin, SAU; 7 College of Medicine, Arabian Gulf University, Manama, BHR; 8 College of Medicine, King Abdulaziz University, Jeddah, SAU; 9 College of Medicine, Al-Rayan Colleges, Madinah Al-Munawwarah, SAU

**Keywords:** ergonomic practices, cervical spine disorders, musculoskeletal disorders, smartphone overuse, neck pain

## Abstract

Background: Smartphone usage has become ubiquitous in Saudi Arabia with concerns growing over its impact on musculoskeletal health. Globally, various musculoskeletal symptoms have been linked to smartphone use such as neck pain, headaches, and shoulder discomfort, often exacerbated by poor posture and prolonged screen time.

Methods: This cross-sectional study was conducted in Riyadh, Saudi Arabia, to investigate the impact of smartphone use on musculoskeletal health among adults aged 18 years and older. Participants were recruited through convenience sampling from various settings such as universities, workplaces, and public areas. Data were collected using a structured questionnaire administered both online and in paper format, covering demographic characteristics, smartphone usage behaviors, awareness of smartphone-related health effects, and the prevalence and severity of musculoskeletal symptoms.

Results: A total of 413 participants from Riyadh, Saudi Arabia, were surveyed to assess musculoskeletal symptoms associated with smartphone use. Demographic analysis revealed a predominantly young, single, and highly educated population, primarily Saudi nationals. Smartphone usage patterns showed extensive daily use, with over 41% using their devices for more than five hours daily. The majority were aware of smartphone-related health effects. Musculoskeletal symptoms were prevalent, notably neck pain (83.8%), arm pain (63.8%), and headaches (71.2%). The incidence of symptoms related to text neck syndrome was substantial, although awareness and diagnosis were limited. The severity of symptoms varied, with mild to moderate levels reported most frequently.

Conclusion: This study highlights a substantial prevalence of musculoskeletal symptoms among smartphone users in Riyadh, Saudi Arabia, emphasizing the need for public health interventions to promote ergonomic practices and mitigate health risks associated with smartphone use. Future research should focus on longitudinal studies to establish causal relationships and evaluate intervention strategies aimed at reducing these symptoms effectively.

## Introduction

The widespread adoption of smartphones has revolutionized communication and access to information globally. In Saudi Arabia, as in many countries, smartphone penetration is exceptionally high, with a significant proportion of the population relying extensively on these devices for daily tasks, communication, and entertainment [1]. While smartphones offer undeniable convenience, prolonged usage has raised concerns regarding their potential health impacts, particularly on musculoskeletal health [2].

Studies globally have increasingly reported a range of musculoskeletal symptoms associated with smartphone use, often referred to collectively as smartphone-related musculoskeletal disorders [3]. These symptoms commonly include neck pain, headaches, shoulder pain, and hand numbness, attributed to poor posture during smartphone use, repetitive movements, and prolonged periods of device interaction. Such issues are exacerbated by the inclination to tilt the head forward and downward while using smartphones, placing increased strain on the cervical spine and upper extremities [4].

In Saudi Arabia, a country where smartphone usage is integrated deeply into daily life across all age groups, understanding the prevalence and impact of smartphone-related musculoskeletal disorders is crucial for informing public health strategies [5]. Despite the growing recognition of these issues internationally, there remains a paucity of research specifically addressing smartphone-related musculoskeletal disorders among Saudi Arabian adults [6], particularly in regions such as Riyadh.

This study aims to fill this gap by investigating the prevalence of musculoskeletal symptoms associated with smartphone use among adults in Riyadh, Saudi Arabia. By identifying the extent and nature of these symptoms, this study aims to provide insights into the public health implications and inform targeted interventions to promote ergonomic practices and mitigate health risks associated with smartphone use in the region.

## Materials and methods

Study design and participants

This was a cross-sectional study conducted in Riyadh, Saudi Arabia, to assess the impact of smartphone use on musculoskeletal health. The study was approved by the Jazan University Research Ethics Committee. The study population comprised individuals aged 18 years and older who were smartphone users. Participants were recruited using a convenience sampling method from various locations, including universities, workplaces, and public areas.

Data collection

Data were collected using a structured questionnaire (See Appendices), distributed both online and in paper form. The questionnaire gathered information on demographic characteristics, smartphone usage patterns, awareness of the effects of smartphone use, and the prevalence and severity of musculoskeletal symptoms. The questionnaire was pre-tested in a small group to ensure clarity and relevance.

Variables and measurements

Primary variables included demographic information (gender, age, marital status, education level, and nationality), smartphone use (daily usage duration and typical neck position while using a smartphone), awareness of the effects of smartphone use on health, and the prevalence and severity of symptoms (neck pain, arm pain, shoulder pain, upper back pain, headaches, stiff neck, and hand numbness). Symptoms were measured using a Likert scale ranging from "strongly disagree" to "strongly agree". Additional variables related to "text neck" syndrome included awareness, sources of information, diagnosis, and associated symptoms and severity.

Data analysis

Data analysis was done with IBM SPSS Statistics for Windows, Version 27.0 (Released 2020; IBM Corp., Armonk, New York, United States). Descriptive statistics were used to summarize demographic characteristics, smartphone use patterns, and prevalence of symptoms. Frequencies and percentages were calculated for categorical variables, while means and standard deviations were used for continuous variables.

## Results

Demographic characteristics

A total of 413 participants were surveyed, with the demographic characteristics presented in Table 1. The sample comprised 57.6% females (n=238) and 42.4% males (n=175). The majority of participants were within the age range of 18-25 years (46.7%, n=193), followed by those aged 26-30 years (16.0%, n=66) and 31-40 years (13.3%, n=55). Most participants were single (60.5%, n=250) and held a college degree (74.8%, n=309). The vast majority were Saudi nationals (97.3%, n=402).

**Table 1:** Demographic characteristics of the sample (N = 413)

Characteristic	Category	Frequency	Percentage
Age Group (years)	Under 18	15	3.6
	18-25	193	46.7
	26-30	66	16.0
	31-40	55	13.3
	41-50	40	9.7
	Above 50	44	10.7
Gender	Male	175	42.4
	Female	238	57.6
Marital Status	Single	250	60.5
	Married	145	35.1
	Divorced	17	4.1
	Widowed	1	0.2
Education Level	Primary	1	0.2
	Intermediate	10	2.4
	High School	75	18.2
	College	309	74.8
	Higher Studies	18	4.4
Nationality	Saudi	402	97.3
	Non-Saudi	11	2.7

Smartphone use and awareness

As shown in Table 2, daily smartphone use varied among participants, with 41.6% (n=172) using their smartphones for more than five hours per day and 37.8% (n=156) for three to five hours. The predominant neck position while using smartphones was 30 degrees (45.8%, n=189), followed by 15 degrees (19.4%, n=80). A significant majority (87.9%, n=363) were aware of the potential effects of smartphone use on their health.

**Table 2:** Smartphone use and awareness (N = 413)

Variable	Category	Frequency	Percentage
Daily Smartphone Use	0-2 hours	16	3.9
	2-3 hours	69	16.7
	3-5 hours	156	37.8
	More than 5 hours	172	41.6
Neck Position	0 degrees	15	3.6
	15 degrees	80	19.4
	30 degrees	189	45.8
	45 degrees	104	25.2
	60 degrees	25	6.1
Awareness of Smartphone Effects	No	50	12.1
	Yes	363	87.9

Symptoms related to smartphone use

The survey results indicated a high prevalence of musculoskeletal symptoms among participants, as detailed in Table 3. Neck pain was reported by 83.8% (n=346) of participants, with 46.5% agreeing and 37.3% strongly agreeing that they experienced neck pain. Arm pain was also common, with 41.9% agreeing and 21.1% strongly agreeing. The presence of shoulder pain was agreed by 42.1% and strongly agreed by 20.6%. Upper back pain, headaches, and stiff neck were similarly prevalent, with a notable percentage of participants indicating they experienced these symptoms.

**Table 3:** Symptoms related to smartphone use (N = 413)

Symptom	Category	Frequency	Percentage
Neck Pain	Strongly Disagree	28	6.8
	Disagree	15	3.6
	Neutral	24	5.8
	Agree	192	46.5
	Strongly Agree	154	37.3
Arm Pain	Strongly Disagree	37	9.0
	Disagree	36	8.7
	Neutral	80	19.4
	Agree	173	41.9
	Strongly Agree	87	21.1
Shoulder Pain	Strongly Disagree	36	8.7
	Disagree	30	7.3
	Neutral	88	21.3
	Agree	174	42.1
	Strongly Agree	85	20.6
Upper Back Pain	Strongly Disagree	29	7.0
	Disagree	30	7.3
	Neutral	75	18.2
	Agree	182	44.1
	Strongly Agree	97	23.5
Headaches	Strongly Disagree	27	6.5
	Disagree	24	5.8
	Neutral	63	15.3
	Agree	176	42.6
	Strongly Agree	123	29.8
Stiff Neck	Strongly Disagree	34	8.2
	Disagree	44	10.7
	Neutral	58	14.0
	Agree	169	40.9
	Strongly Agree	108	26.2
Hand Numbness	Strongly Disagree	33	8.0
	Disagree	41	9.9
	Neutral	79	19.1
	Agree	173	41.9
	Strongly Agree	87	21.1

Incidence of pain and awareness of text neck syndrome

As outlined in Table 4, 81.1% (n=335) of participants experienced neck pain, and 68.0% (n=281) reported neck pain specifically related to smartphone use. Arm pain was reported by 42.6% (n=176), shoulder pain by 49.4% (n=204), and upper back pain by 42.6% (n=176). Headaches were experienced by 63.0% (n=260), and 24.9% (n=103) had a stiff neck. Furthermore, 32.9% (n=136) reported hand numbness. Awareness of text neck syndrome was low, with only 16.9% (n=70) having heard of it, predominantly from the internet (15.0%, n=62). Only 4.8% (n=20) had been diagnosed with text neck.

**Table 4:** Incidence of pain and awareness of text neck syndrome (N = 413)

Variable		Frequency	Percentage
Had Neck Pain		335	81.1
Had Arm Pain		176	42.6
Had Shoulder Pain		204	49.4
Had Upper Back Pain		176	42.6
Had Headaches		260	63.0
Had Stiff Neck		103	24.9
Had Hand Numbness		136	32.9
Neck Pain While Using Smartphone		281	68.0
Heard of Text Neck Syndrome		70	16.9
Source of Text Neck Information	Friends/Relatives	22	5.3
	Internet	62	15.0
	Doctors	4	1.0
	Medical Journals	17	4.1
	Not Sure	308	74.6
Diagnosed with Text Neck Syndrome	No	393	95.2
	Yes	20	4.8

The severity of neck pain and associated symptoms

Table 5 presents the severity of neck pain and associated symptoms. Mild neck pain was the most commonly reported (63.2%, n=261), followed by severe pain (9.0%, n=37) and very severe pain (4.6%, n=19). Regarding headaches associated with smartphone use, 38.5% (n=159) experienced little headaches, 24.5% (n=101) had moderate headaches, and 9.0% (n=37) had severe headaches. Stiff neck frequency varied, with 32.2% (n=133) experiencing it occasionally, 23.2% (n=96) rarely, and 16.0% (n=66) frequently. Upper back pain was also notable, with 28.1% (n=116) experiencing it occasionally and 16.2% (n=67) frequently.

**Table 5:** Severity of neck pain and associated symptoms (N = 413)

Variable	Category	Frequency	Percentage
Severity of Neck Pain	No Pain	95	23.0
	Mild	261	63.2
	Severe	37	9.0
	Very Severe	19	4.6
	Intolerable	1	0.2
Headaches with Smartphone Use	No Headache	110	26.6
	Little	159	38.5
	Moderate	101	24.5
	Severe	37	9.0
	Very Severe	6	1.5
Stiff Neck Frequency	Never	95	23.0
	Rarely	96	23.2
	Occasionally	133	32.2
	Frequently	66	16.0
	Always	23	5.6
Upper Back Pain Frequency	Never	130	31.5
	Rarely	77	18.6
	Occasionally	116	28.1
	Frequently	67	16.2
	Always	23	5.6

## Discussion

This study highlights the significant prevalence of musculoskeletal symptoms among smartphone users in Riyadh, Saudi Arabia. The findings indicate a high incidence of neck pain, with 81.1% of participants reporting experiencing this symptom. Other common complaints included headaches (63.0%), hand numbness (32.9%), and shoulder pain (49.4%). These results align with existing literature that associates prolonged smartphone use with musculoskeletal discomfort and pain [7-9].

Our findings are consistent with previous studies conducted in various regions, which have documented similar patterns of musculoskeletal symptoms linked to smartphone use. For instance, a study found a high prevalence of neck and shoulder pain among smartphone users, particularly those who used their devices for extended periods and maintained poor postures [10]. Similarly, studies have reported increased incidences of text neck syndrome and associated musculoskeletal issues among heavy smartphone users [10,11].

The high prevalence of musculoskeletal symptoms observed underscores the need for public health interventions aimed at mitigating the adverse effects of smartphone use [12]. Educational campaigns to raise awareness about proper smartphone ergonomics and posture could be beneficial [13]. Additionally, incorporating regular breaks and exercises to alleviate tension and strain may help reduce the incidence of these symptoms [14,15].

Future studies should consider longitudinal designs to better understand the causal relationships between smartphone use and the development of musculoskeletal symptoms. It would also be valuable to explore the effectiveness of various interventions aimed at reducing these symptoms among smartphone users. Expanding the research to include diverse populations and settings could provide a more comprehensive understanding of this public health issue.

This study has several strengths, including a comprehensive assessment of a wide range of musculoskeletal symptoms and the use of a structured questionnaire to ensure systematic data collection. The sample size was adequate, providing a robust dataset for analysis. However, some limitations should be acknowledged. The use of convenience sampling may limit the generalizability of the findings to the broader population. Additionally, the cross-sectional design precludes the ability to establish causality between smartphone use and musculoskeletal symptoms.

## Conclusions

This study underscores the widespread prevalence of musculoskeletal symptoms associated with smartphone use among adults. Neck pain emerged as the predominant issue, followed by headaches, shoulder pain, and hand numbness. These findings emphasize the urgent need for public health interventions aimed at promoting ergonomic practices and raising awareness about the potential health risks of prolonged smartphone use. Implementing strategies such as educational campaigns on proper posture and regular breaks could help mitigate these symptoms. Further research is warranted to explore longitudinal impacts and evaluate the effectiveness of interventions in reducing musculoskeletal discomfort among smartphone users.

## Appendices

Survey questionnaire

Section 1: Demographic Information

1. Age: Under 18 / 18-25 / 26-30 / 31-40 / 41-50 / Above 50

2. Gender: Male / Female

3. Marital Status: Single / Married / Divorced / Widowed

4. Education Level: Primary / Intermediate / High School / College / Higher Studies

5. Nationality: Saudi / Non-Saudi

Section 2: Smartphone Use

6. Daily Smartphone Use: 0-2 hours / 2-3 hours / 3-5 hours / More than 5 hours

7. Typical Neck Position: 0 degrees / 15 degrees / 30 degrees / 45 degrees / 60 degrees

8. Aware of Health Effects: Yes / No

Section 3: Musculoskeletal Symptoms

9. Neck Pain: Strongly Disagree / Disagree / Neutral / Agree / Strongly Agree

10. Arm Pain: Strongly Disagree / Disagree / Neutral / Agree / Strongly Agree

11. Shoulder Pain: Strongly Disagree / Disagree / Neutral / Agree / Strongly Agree

12. Upper Back Pain: Strongly Disagree / Disagree / Neutral / Agree / Strongly Agree

13. Headaches: Strongly Disagree / Disagree / Neutral / Agree / Strongly Agree

14. Stiff Neck: Strongly Disagree / Disagree / Neutral / Agree / Strongly Agree

15. Hand Numbness: Strongly Disagree / Disagree / Neutral / Agree / Strongly Agree

Section 4: Incidence and Awareness of Text Neck Syndrome

16. Experienced Neck Pain: Yes / No

17. Experienced Arm Pain: Yes / No

18. Experienced Shoulder Pain: Yes / No

9. Experienced Upper Back Pain: Yes / No

20. Experienced Headaches: Yes / No

21. Experienced Stiff Neck: Yes / No

22. Experienced Hand Numbness: Yes / No

23. Neck Pain While Using Smartphone: Yes / No

24. Heard of Text Neck Syndrome: Yes / No

25. Source of Text Neck Information (select all that apply): Friends/Relatives / Internet / Doctors / Medical Journals / Not Sure

26. Diagnosed with Text Neck Syndrome: Yes / No

Section 5: Severity of Symptoms For Each Symptom, Indicate the Severity:

27. Severity of Neck Pain: No Pain / Mild / Severe / Very Severe / Intolerable

28. Severity of Headaches with Smartphone Use: No Headache / Little / Moderate / Severe / Very Severe

29. Frequency of Stiff Neck: Never / Rarely / Occasionally / Frequently / Always

30. Frequency of Upper Back Pain: Never / Rarely / Occasionally / Frequently / Always
